# Body mass index is associated with risk of reoperation and revision after primary total hip arthroplasty: a study of the Swedish Hip Arthroplasty Register including 83,146 patients

**DOI:** 10.1080/17453674.2019.1594015

**Published:** 2019-04-01

**Authors:** Arkan S Sayed-Noor, Sebastian Mukka, Maziar Mohaddes, Johan Kärrholm, Ola Rolfson

**Affiliations:** a Department of Surgical and Perioperative Sciences, Umeå University, Umeå;;; b Swedish Hip Arthroplasty Register, Centre of Registers, Gothenburg;;; c Department of Orthopaedics, Institute of Clinical Sciences, Sahlgrenska Academy, University of Gothenburg, Gothenburg, Sweden

## Abstract

Background and purpose — The prevalence of obesity is on the rise, becoming a worldwide epidemic. The main purpose of this register-based observational study was to investigate whether different BMI classes are associated with increased risk of reoperation within 2 years, risk of revision within 5 years, and the risk of dying within 90 days after primary total hip arthroplasty (THA). We hypothesized that increasing BMI would increase these risks.

Patients and methods — We analyzed a cohort of 83,146 patients who had undergone an elective THA for primary osteoarthritis between 2008 and 2015 from the Swedish Hip Arthroplasty Register (SHAR). BMI was classified according to the World Health Organization (WHO) into 6 classes: < 18.5 as underweight, 18.5–24.9 as normal weight, 25–29.9 as overweight, 30–34.9 as class I obesity, 35–39.9 as class II obesity, and ≥ 40 as class III obesity.

Results — Both unadjusted and adjusted parameter estimates showed increasing risk of reoperation at 2 years and revision at 5 years with each overweight and obesity class, mainly due to increased risk of infection. Uncemented and reversed hybrid fixations and surgical approaches other than the posterior were all associated with increased risk. Obesity class III (≥ 40), male sex, and increasing ASA class were associated with increased 90-day mortality.

Interpretation — Increasing BMI was associated with 2-year reoperation and 5-year revision risks after primary THA where obese patients have a higher risk than overweight or normal weight patients. As infection seems to be the main cause, customizing preoperative optimization and prophylactic measures for obese patients may help reduce risk.

The prevalence of obesity is on the rise, becoming a worldwide epidemic. Currently, more than two-thirds of Americans are classified as obese (Yang and Colditz 2015). In obese patients, total hip arthroplasty (THA) can be challenging because the extensive adipose tissue can compromise optimal surgical technique, prolong operative time, and increase intraoperative bleeding and risk for postoperative complications (Bowditch and Villar 1999, Liu et al. 2015, Wooten and Curtin 2016, Krauss et al. 2018). The effect of BMI on functional outcome, quality of life, and complication rate following THA has been investigated in a number of studies (Vincent et al. 2012, Liu et al. 2015, Haynes et al. 2017, Barrett et al. 2018). As BMI increases, the functional improvement and quality of life after THA may deteriorate. Based on this presumed increased risk, the American Association of Hip and Knee Surgeons Workgroup released a statement recommending that arthroplasty operations in patients with a BMI > 40 be delayed, especially in the setting of other comorbid conditions (Workgroup of the American Association of Hip and Knee Surgeons Evidence Based Committee 2013). However, only a limited number of studies reporting perioperative complications have been based on population-based cohorts (Murgatroyd et al. 2014, Ward et al. 2015, Husted et al. 2016, Wagner et al. 2016, Jung et al. 2017, Werner et al. 2017, Zusmanovich et al. 2018, DeMik et al. 2018, Jeschke et al. 2018).

The main purpose of this register-based cohort study was to investigate whether under- or overweight, separated into BMI classes, is associated with increased risk of reoperation within 2 years, risk of revision within 5 years, and the risk of dying within 90 days after primary total hip arthroplasty. We hypothesized that increasing BMI would negatively influence the reoperation, revision, and mortality risks. 

## Patients and methods

### Study design and setting

The Swedish Hip Arthroplasty Register (SHAR) was launched in 1979 to prospectively monitor THAs performed in Sweden and to evaluate the performance of implants, fixation methods, and surgical techniques. The register covers all publicly and privately funded hospitals performing THA. The completeness of registration for primary THAs is between 97% and 99%. A unique patient identifier, the personal identity number, provides information on date of birth and sex. For each operation, participating hospitals record variables such as implant article number, type of fixation, and surgical approach. In 2008, information on American Association of Anesthesiologists’ physical status classification (ASA), weight, and height were added to the routine data collection.

We followed the STROBE guidelines (von Elm et al. 2014).

### Patient selection

Patients included in this observational study met the following criteria: primary osteoarthritis (International Classification of Diseases [ICD] M16.0 and M16.1) operated with THA between January 1, 2008 and December 31, 2015 using traditional (not resurfacing) implants with uncemented, cemented, hybrid, or reversed hybrid fixation. In patients with bilateral THA during the study period, we included only records concerning the first THA. Patients with missing documentation regarding BMI or ASA class were excluded. BMI was classified according to the World Health Organization (WHO) into 6 classes: < 18.5 as underweight, 18.5–24.9 as normal weight, 25–29.9 as overweight, 30–34.9 as class I obesity, 35.0–39.9 as class II obesity, and ≥ 40 as class III obesity.

### Outcome measures

Reoperation is defined as any kind of subsequent open surgical procedure related to the inserted arthroplasty, no matter whether the arthroplasty, or any of its parts, is replaced, extracted, or left untouched. Revision is defined as a subsequent procedure where at least 1 part of the prosthesis is exchanged, added to, or extracted. All revisions are also classified as reoperations, but not all reoperations are revisions.

The outcome measures of this study include:

1. Reoperations within the first 2 years from the index THA procedure, including all types of open surgical procedures to the hip and for any reason;
2. Revisions within the first 5 years from the index THA procedure. For first-time procedures, a revision in the SHAR is defined as exchange or removal of one or more implant component(s);
3. 90-day mortality. The mortality data are obtained by cross-matching data from SHAR with the Swedish Population Register, governed by the Swedish Tax Office.

Causes of reoperation and revision were categorized into loosening/osteolysis, dislocation, infection, and other.

### Confounders

A priori, we decided to include the age, sex, ASA class, fixation method, and surgical approach as confounders. These variables have previously demonstrated association with both exposure and outcome and are not considered to be in the causal pathway between potential risk factors and outcome.

### Statistics

Survival estimates (with 95% confidence intervals [CI]) for not being reoperated within 2 years, not revised within 5 years, and being alive within 90 days were calculated using Kaplan–Meier survival analysis. The assumption of proportionality was checked graphically. Simple and multiple Cox regression analyses were applied to calculate unadjusted and adjusted hazard ratios (HR). We adjusted for age, sex, ASA class, fixation method, and surgical approach at primary surgery. R version 3.4.4 (https://www.r-project.org) was used to perform all analyses.

### Ethics, funding, and potential conflicts of interests

The study was conducted in accordance with the ethical principles of the Helsinki Declaration and was approved by the Regional Ethical Review Board in Gothenburg, Sweden (decision 271-14). There was no external funding for the project and no competing interest to declare.

## Results

**Figure 1.**
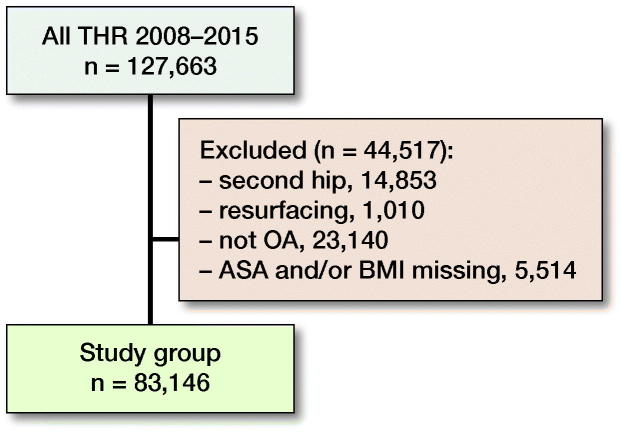
Flowchart of patients through the study.

127,663 primary THAs, registered in SHAR between January 1, 2008 and December 31, 2015 were primarily included. After exclusion of resurfacing arthroplasties, second hip THA, patients with secondary OA, and those with missing data, 83,146 patients (mean age 69 years, 57% females) remained for analysis (Figure 1, Table 1). The majority of patients were normal weight or overweight. Age at operation decreased and the ASA class increased with increasing weight and obesity class. The dominating fixation technique was cemented and a posterior approach was used in nearly half of the operations.

**Table 1.** Demography per BMI class

Factor	Underweight	Normal weight	Overweight	Class I obesity	Class II obesity	Class III obesity	All patients
Number of patients	579 (	25,718 (	36,301 (	15,751 (	3,939 (	858 (	83,146 (
Age, mean (SD)	73 (11)	70 (10)	69 (10)	67 (9)	65 (9)	64 (9)	69 (10)
Female sex, n (%)	516 (89)	16,721 (65)	18,360 (51)	8,568 (54)	2,455 (62)	590 (69)	47,210 (57)
ASA, n (%)							
I	125 (22)	7,998 (31)	9,147 (25)	2,499 (16)	272 (6.9)	54 (6)	20,095 (24)
II	330 (57)	14,519 (56)	22,356 (62)	10,120 (64)	2,224 (57)	391 (46)	49,940 (60)
III	116 (20)	3,120 (12)	4,675 (13)	3,067 (19)	1,416 (36)	398 (46)	12,792 (15)
IV/V	8 (1)	81 (0.3)	123 (0.3)	65 (0.4)	27 (1)	15 (2)	319 (0.4)
Fixation, n (%)							
All cemented	464 (80)	18,146 (71)	24,342 (67)	10,359 (66)	2,532 (64)	539 (63)	56,382 (68)
All uncemented	46 (8)	3,839 (15)	6,342 (17)	2,890 (18)	775 (20)	186 (22)	14,078 (17)
Hybrid	16 (3)	562 (2)	676 (2)	281 (2)	65 (2)	20 (2)	1,620 (2)
Reversed hybrids	53 (9)	3,171 (12)	4,941 (14)	2,221 (14)	567 (14)	113 (13)	11,066 (13)
Surgical approach, n (%)							
Posterior	273 (47)	13,044 (51)	19,049 (53)	8,496 (54)	2,119 (54)	477 (56)	43,458 (52)
Direct lateral	253 (44)	10,859 (42)	15,046 (41)	6,435 (41)	1,643 (42)	353 (41)	34,589 (42)
Other	53 (9)	1,813 (7)	2,205 (6)	818 (5)	177 (5)	28 (3)	5,094 (6)

Table 2 (see Supplementary data) presents survival estimates at 2 years for reoperation, 5 years for revision, and 90 days for mortality among the 6 BMI classes.

### Risk of reoperation within 2 years

**Figure 2.**
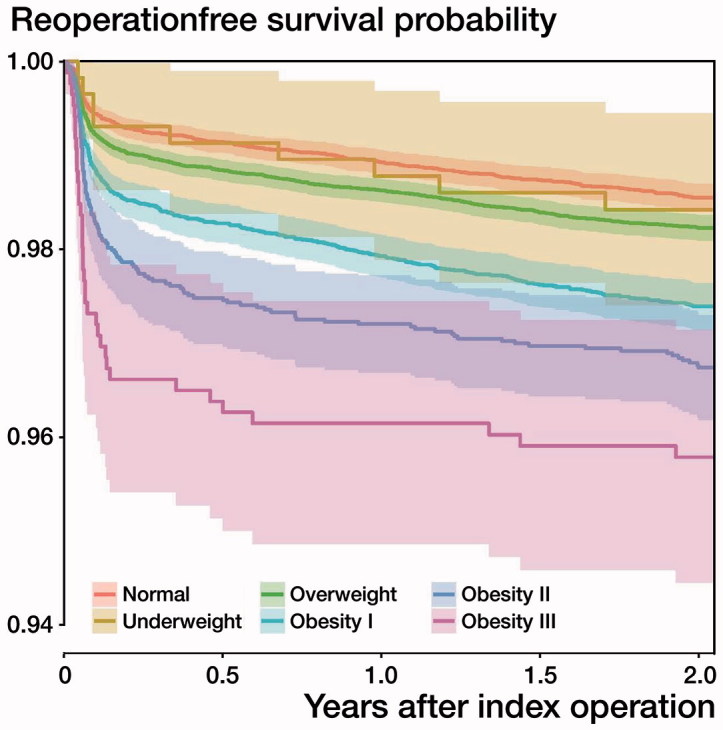
Kaplan–Meier 2-year reoperation estimates by BMI class (including CIs).

The probability of reoperation increased in overweight and obesity classes I–III (Figure 2). Both unadjusted and adjusted parameter estimates showed increasing risk of reoperation at 2 years with each overweight and obesity class, mainly due to increased risk of infection, whereas the HR for underweight was similar to the reference category normal weight (Tables 2 and 3, see Supplementary data). The 2-year risk of reoperation was higher in men and increased with higher ASA class. Uncemented fixation and reversed hybrid fixations, and other surgical approaches than the posterior were all associated with increased risk.

### Risk of revision within 5 years

**Figure 3.**
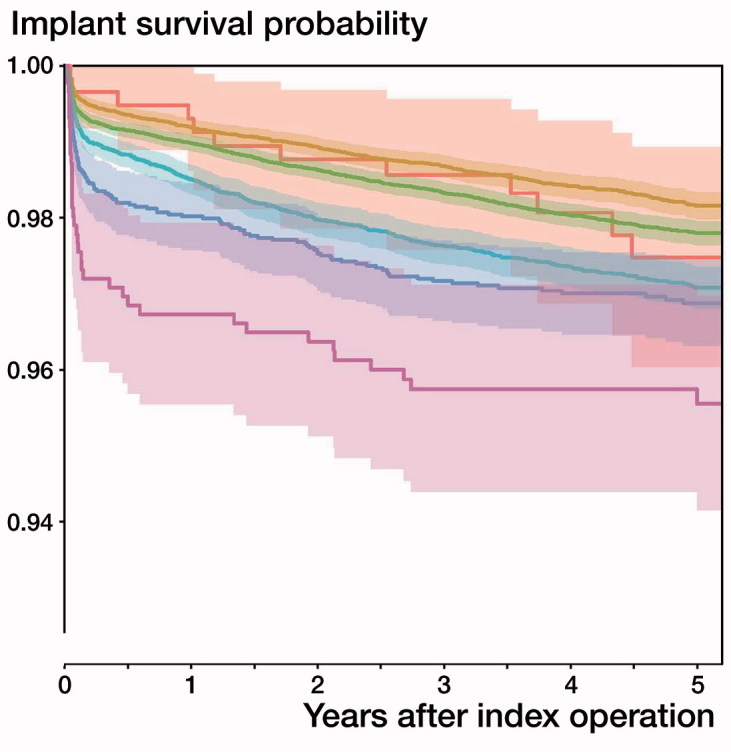
Kaplan–Meier 5-year implant survival estimates by BMI class (including CIs). For color codes, see Figure 2.

The probability of not being revised was lower in BMI overweight and obesity classes I–III (Figure 3). Both unadjusted and adjusted parameter estimates showed increasing risk of revision at 5 years with each overweight and obesity class, mainly due to increased risk of infection, while the HR for underweight was similar to the reference category normal weight (Tables 2 and 3, see Supplementary data). The 5-year risk of revision was higher in men and increased with higher ASA class. Uncemented fixation and reversed hybrid fixations, and other surgical approaches than the posterior were all associated with increased risk.

### 90-day mortality

**Figure 4.**
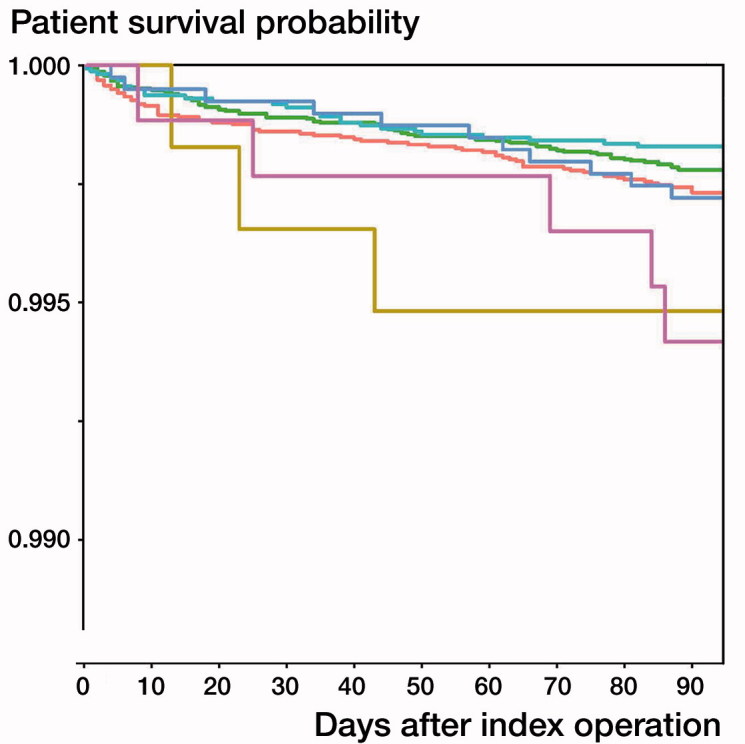
Kaplan–Meier 90-day mortality estimates by BMI class. For color codes, see Figure 2.

Underweight and obesity class III were associated with higher mortality compared with the other BMI classes (Figure 4). However, HRs for BMI classes were not statistically significantly higher compared with normal weight (Table 3, see Supplementary data). In the multiple regression model, only obesity class III (≥ 40), male sex, and increasing ASA class were associated with increased risk.

## Discussion

The impact of bodyweight on the occurrence and progression of hip OA as well as on the early and late results of THA has been in focus during the last 2 decades. Several studies have shown that overweight may be associated with hip OA symptoms, motivating THA at early ages (Harms et al. 2007, Changulani et al. 2008, Gandhi et al. 2010). Furthermore, increased BMI has been linked to higher risks for perioperative complications such as bleeding, infection, and dislocation, even though there is still a debate over the validity of some of these results (Vincent et al. 2012, Liu et al. 2015, Haynes et al. 2017, Barrett et al. 2018). The concept of obesity paradox, i.e., the favourable and protective effect of obesity on some aspects of the outcome of THA, has been raised (Shaparin et al. 2016, Zhang et al. 2018).

By definition, obesity class I and II would yield an ASA class of II or above and obesity class III would yield ASA class III or above. Although there was a clear pattern with higher ASA class for the obesity levels, not all patients were classified as per the definition. This highlights the interrater variation in assessment of ASA class and possible local traditions among anesthesiologists in how the ASA classification system is applied (Sankar et al. 2014). Nevertheless, we believe this also reflects the result of an overall assessment of perioperative risk factors where some otherwise healthy obese patients are classified lower than as defined by the classification system.

In our study, patients with class III obesity were younger. This concurs with other studies (Harms et al. 2007, Changulani et al. 2008, Gandhi et al. 2010). Changulani et al. (2008) studied the relationship between obesity and age among 1,025 THA patients and found that the morbidly obese were 10 years younger on average than those with a normal BMI. In their systematic review, Haynes et al. (2017) also found that obesity was associated with younger age at time of primary THA. These findings agree with a review of registry data from the Mayo Clinic (Singh and Lewallen 2014), which showed a decrease in the mean age of patients undergoing primary THA by 0.7 years. This was inversely associated with an increase of 1.6 in the BMI of primary THA patients over the same study period (1993–2005). A possible explanation for this association might be the increased pain sensitivity, high-level forces/wear on the joint surface, and the lower physical activity in morbidly obese patients.

We found an association of BMI class with increasing risk of reoperation within 2 years and revision within 5 years, mainly due to increased risk of infection (Table 2). This concurs with a recent report using German nationwide billing data for inpatient hospital treatment covering more than 130,000 THAs. In this report, Jeschke et al. (2018) found increased overall postoperative complication and 1-year revision rates with higher BMI class. Similar to our results, they found that 90-day mortality increased only in class III obesity patients. Other studies have demonstrated increased postoperative infection and dislocation rates both after primary and revision THA with increasing BMI (Vincent et al. 2012, Pulos et al. 2014, Houdek et al. 2015, Liu et al. 2015, Haynes et al. 2017, Barrett et al. 2018, Kennedy et al. 2018). Contrary to the above-mentioned results, some reports showed comparable postoperative complication rates across BMI classes (Davis et al. 2011, McCalden et al. 2011, Watts et al. 2016). This divergence requires further attention and analysis. Increased BMI is associated or has a causal relationship with medical comorbidities such as diabetes mellitus, and cardiovascular disorders, as well as antibiotic resistance. Moreover, THA in obese patients may be more surgically demanding as the voluminous deep adipose tissue, weak fatty-infiltrated peri-articular soft-tissue envelope, and obscured anatomical landmarks may result in suboptimal positioning of THA components, prolonged operative time, and wound problems (Elson et al. 2013, Hanly et al. 2016). Higher weight increases load and forces on THA components, which potentially increases the risk of wear and implant loosening. However, a sedentary lifestyle might counteract this risk for wear and aseptic loosening. The above-mentioned parameters may, at least partly, explain the negative impact of increased BMI on 2-year reoperation and 5-year revision outcomes. Interestingly, we found comparable risks for reoperation within 2 years and revision within 5 years due to mechanical complications (loosening and dislocation) among the BMI classes (Table 2, see Supplementary data). Patients with increased BMI may also have some positive aspects such as adequate nutrition, careful preoperative preparation and surgical technique usually performed by more experienced surgeons, more active postoperative medical care, and physical rehabilitation and follow-up. These aspects can be protective to some extent, but apparently not for patients with a very high BMI such as class III obesity.

This study has some limitations. SHAR does not capture all reoperations. The completeness of registrations of revisions is higher than that of other reoperations where components are not removed, exchanged, or added. Also, there are missing data in the reporting of weight and height. There is no reason to suspect a systematic underreporting of BMI or reoperations based on BMI. However, the most common cause for a reoperation without revising implants is periprosthetic infection. Given that high BMI is a risk factor for infection, the underreporting may distribute differently between BMI classes. Hence, the higher risk of reoperation associated with increasing BMI class may be underestimated. Despite the comprehensive set of variables included in SHAR, parameters such as smoking, type of comorbidities, nutritional status, OA severity, surgical complexity, and surgeon experience were not available. Therefore, as with most register-based studies, residual confounding likely exists. Also, we did not correct for multiple testing; however, confounders were selected a priori and based on previous established relationships. The methods for measuring weight and height are heterogeneous and include estimates by health care professionals, patient-reported values, and actual measurements at the preoperative assessment. Another limitation pertains to the use of BMI as a surrogate measure for excess fat although it does not distinguish between the distributions of fat, muscles, and bone mass. On average, women have greater amounts of total body fat than men with an equivalent BMI, while muscular highly trained athletes may have a high BMI because of increased muscle mass. Furthermore, there are numerous factors related to genetics, and the physical and social environment including comorbidities that will influence the body mass index. Thus, BMI could be viewed as a proxy for known and unknown factors related to the health status of the patient, but is as such attractive to use because it can be easily measured. Multiple comparisons among the BMI classes is another limitation. These limitations are counterbalanced by the strength of study: a nationwide large study group using a register with high completeness and validity. The inclusion of not only revisions as an endpoint but also any reoperation adds to the strength of the study.

In summary, BMI classes were associated with reoperation and revision risks after primary THA, where morbidly obese patients have more than a doubled risk than obese or normal weight patients. As infection seems to be the main cause, customizing preoperative optimization and prophylactic measures for obese patients may help reduce risk. Furthermore, many clinical aspects could be addressed such as adequate antibiotic prophylaxis, e.g., weight-adjusted as well as the use of incisional vacuum-assisted closure in overweight patients. Although BMI is a well-established risk factor for complications following THA, this is the first study focusing on BMI and outcomes in a Swedish context. This will help inform surgeons and their patients on risks related to BMI classes.

### Supplementary data

Tables 2–3 are available as supplementary data in the online version of this article, http://dx.doi.org/10.1080/17453674. 2019.1594015

ASN and OR conceived the study and defined the analysis plan with input from all other co-authors. ASN, OR, and SM drafted the manuscript. All authors interpreted results and reviewed the manuscript.

The authors would like to thank all orthopedic surgeons and administrative personnel at Swedish hospitals for their contribution of data and engagement in the register. They also thank registry coordinator Pär Werner who performed statistical analyses.

*Acta* thanks Jeppe Lange and Mogens Laursen for help with peer review of this study.

## Supplementary data

## Supplementary Material

Supplementary Material
