# Supplementary material for: Body mass index is associated with risk of reoperation and revision after primary total hip arthroplasty: a study of the Swedish Hip Arthroplasty Register including 83,146 patients
Source: Acta Orthop. 2019 Apr 1;90(3):220–5. doi: 10.1080/17453674.2019.1594015 (PMC6534237; doi:10.1080/17453674.2019.1594015)
Supplement: Supplementary Material [file IORT_A_1594015_SM6605.pdf]

Supplementary data

Table 2. Kaplan–Meier estimates per BMI class

Factor	Underweight	Normal weight	Overweight	Class I obesity	Class II obesity	Class III obesityAll patients
Not reoperated within 2 years (95% CI)						
All causes	0.984 (0.974–0.994)	0.985 (0.984–0.987)	0.982 (0.981–0.984)	0.974 (0.971–0.976)	0.967 (0.962–0.973)	0.958 (0.945–0.971)
Loosening [a]	0.996 (0.991–1.000)	0.999 (0.998–0.999)	0.998 (0.997–0.998)	0.997 (0.997–0.998)	0.997 (0.995–0.999)	0.999 (0.996–1.000)
Dislocation	0.996 (0.991–1.000)	0.997 (0.997–0.998)	0.997 (0.997–0.998)	0.995 (0.994–0.997)	0.996 (0.994–0.998)	0.996 (0.992–1.000)
Infection	0.991 (0.984–0.999)	0.994 (0.993–0.995)	0.991 (0.990–0.992)	0.986 (0.984–0.988)	0.979 (0.975–0.984)	0.970 (0.959–0.982)
Other	1.000 (1.000–1.000)	0.995 (0.994–0.996)	0.996 (0.995–0.997)	0.995 (0.993–0.996)	0.995 (0.992–0.997)	0.992 (0.986–0.998)
5-year implant survival (95% CI)						
All causes	0.975 (0.960–0.989)	0.981 (0.980–0.983)	0.978 (0.976–0.980)	0.971 (0.968–0.974)	0.969 (0.963–0.974)	0.955 (0.942–0.970)
Loosening [a]	0.989 (0.979–0.999)	0.996 (0.995–0.997)	0.994 (0.993–0.995)	0.994 (0.992–0.994)	0.993 (0.991–0.996)	0.996 (0.992–1.000)
Dislocation	0.996 (0.992–1.000)	0.996 (0.995–0.997)	0.995 (0.995–0.996)	0.994 (0.993–0.995)	0.995 (0.993–0.997)	0.994 (0.988–0.999)
Infection	0.992 (0.983–1.000)	0.994 (0.993–0.995)	0.992 (0.991–0.993)	0.987 (0.985–0.989)	0.983 (0.979–0.987)	0.974 (0.963–0.985)
Other	0.998 (0.993–1.000)	0.995 (0.994–0.996)	0.996 (0.995–0.997)	0.996 (0.995–0.997)	0.997 (0.995–0.999)	0.991 (0.984–0.998)
90-day survival (95% CI)						
All	0.995 (0.989–1.000)	0.997 (0.997–0.998)	0.998 (0.997–0.998)	0.998 (0.998–0.999)	0.997 (0.996–0.999)	0.994 (0.989–0.999)
Men	0.984 (0.954–1.000)	0.997 (0.995–0.998)	0.997 (0.996–0.998)	0.997 (0.996–0.998)	0.995 (0.991–0.998)	0.989 (0.976–1.000)
Women	0.996 (0.991–1.000)	0.998 (0.997–0.998)	0.998 (0.998–0.999)	0.999 (0.999–1.000)	0.999 (0.997–1.000)	0.997 (0.992–1.000)

[a] and/or osteolysis

Table 3. Cox regression analyses for reoperation, revision, and mortality

Factor	2-year reoperation HR (95% CI)	5-year revision HR (95% CI)	90-day mortality HR (95% CI)
Unadjusted			
BMI class			
Underweight	1.1 (0.6–2.1)	1.3 (0.7–2.3)	1.9 (0.6–6.1)
Normal weight	1.0 Ref	1.0 Ref	1.0 Ref
Overweight	1.2 (1.1–1.4)	1.2 (1.1–1.4)	0.8 (0.6–1.1)
Class I obesity	1.8 (1.6–2.0)	1.6 (1.4–1.9)	0.6 (0.4–1.0)
Class II obesity	2.3 (1.9–2.7)	1.8 (1.5–2.2)	1.0 (0.6–2.0)
Class III obesity	3.0 (2.1–4.2)	2.6 (1.9–3.7)	2.2 (0.9–5.4)
Adjusted			
BMI class			
Underweight	1.1 (0.6–2.1)	1.4 (0.8–2.5)	1.6 (0.5–5.0)
Normal weight	1.0 Ref	1.0 Ref	1.0 Ref
Overweight	1.2 (1.0–1.3)	1.1 (1.0–1.3)	0.8 (0.6–1.2)
Class I obesity	1.7 (1.5–2.0)	1.5 (1.3–1.7)	0.7 (0.5–1.1)
Class II obesity	2.0 (1.7–2.5)	1.5 (1.2–1.9)	1.1 (0.6–2.2)
Class III obesity	2.7 (1.9–3.8)	2.2 (1.6–3.1)	2.6 (1.0–6.6)
Age–by years	1.0 (1.0–1.0)	1.0 (1.0–1.0)	1.1 (1.1–1.1)
Sex			
Female	1.0 Ref	1.0 Ref	1.0 Ref
Male	1.3 (1.2–1.5)	1.5 (1.3–1.6)	2.2 (1.6–3.0)
ASA class			
I	1.0 Ref	1.0 Ref	1.0 Ref
II	1.3 (1.1–1.5)	1.3 (1.1–1.4)	2.4 (1.3–4.7)
III	1.9 (1.6–2.3)	1.7 (1.4–2.0)	6.1 (3.1–12)
IV/V	2.7 (1.6–4.7)	2.1 (1.2–3.9)	21 (8.2–55)
Fixation			
All cemented	1.0 Ref	1.0 Ref	1.0 Ref
All uncemented	2.0 (1.7–2.3)	2.0 (1.7–2.3)	0.6 (0.3–1.1)
Hybrid	0.4 (0.6–1.4)	1.0 (0.7–1.5)	0.2 (0.03–1.6)
Reversed hybrids	1.5 (1.3–1.8)	1.6 (1.4–1.9)	1.0 (0.6–1.7)
Surgical approach			
Posterior	1.0 Ref	1.0 Ref	1.0 Ref
Direct lateral	1.3 (1.2–1.5)	1.1 (1.0–1.2)	1.2 (0.9–1.7)
Other	1.2 (1.0–1.5)	0.9 (0.8–1.2)	1.2 (0.7–2.2)
